# Opt-Out and Opt-In Testing Increases Syphilis Screening of HIV-Positive Men Who Have Sex with Men in Australia

**DOI:** 10.1371/journal.pone.0071436

**Published:** 2013-08-23

**Authors:** Rebecca Guy, Carol El-Hayek, Christopher K. Fairley, Handan Wand, Andrew Carr, Anna McNulty, Jenny Hoy, Christopher Bourne, John McAllister, B. K. Tee, David Baker, Norman Roth, Mark Stoove, Marcus Chen

**Affiliations:** 1 The Kirby Institute, University of New South Wales, Sydney, New South Wales, Australia; 2 Centre for Population Health, Burnet Institute, Melbourne, Victoria, Australia; 3 Melbourne Sexual Health Centre, Melbourne, Victoria, Australia; 4 Melbourne School of Population Health, University of Melbourne, Melbourne, Victoria, Australia; 5 HIV, Immunology and Infectious Diseases Unit and Centre for Applied Medical Research, St Vincent's Hospital, Sydney, New South Wales, Australia; 6 Sydney Sexual Health Centre, Sydney, New South Wales, Australia; 7 School of Public Health and Community Medicine, University of New South Wales, Sydney, New South Wales, Australia; 8 Infectious Disease Unit, Alfred Hospital, Melbourne, Victoria, Australia; 9 Monash University, Melbourne, Victoria, Australia; 10 The Centre Clinic, Melbourne, Victoria, Australia; 11 East Sydney Doctors, Sydney, New South Wales, Australia; 12 Prahran Market Clinic, Melbourne, Victoria, Australia; Vanderbilt University, United States of America

## Abstract

**Background:**

Since 2005, Australian clinicians were advised to undertake quarterly syphilis testing for all sexually active HIV-positive men who have sex with men (MSM). We describe differences in syphilis testing frequency among HIV-positive MSM by clinic testing policies since this recommendation.

**Methods:**

Three general practices, two sexual health clinics and two hospital HIV outpatient clinics provided data on HIV viral load and syphilis testing from 2006–2010. Men having ≥1 viral load test per year were included; >95% were MSM. We used Chi-2 tests to assess changes in syphilis testing frequency over time, and differences by clinic testing policy (opt-out, opt-in and risk-based).

**Results:**

The proportion of men having HIV viral loads with same-day syphilis tests increased from 37% in 2006 to 63% in 2007 (p<0.01) and 68–69% thereafter. In 2010, same-day syphilis testing was highest in four clinics with opt-out strategies (87%, range:84–91%) compared with one clinic with opt-in (74%, p = 0.121) and two clinics with risk-based strategies (22%, range:20–24%, p<0.01). The proportion of men having ≥3 syphilis tests per year increased from 15% in 2006 to 36% in 2007 (p<0.01) and 36–38% thereafter. In 2010, the proportion of men having ≥3 syphilis tests in a year was highest in clinics with opt-out strategies (48%, range:35–59%), compared with opt-in (39%, p = 0.121) and risk-based strategies (8.4%, range:5.4–12%, p<0.01).

**Conclusion:**

Over five years the proportion of HIV-positive men undergoing syphilis testing at recommended frequencies more than doubled, and was 5–6 times higher in clinics with opt-out and opt-in strategies compared with risk-based policies.

## Introduction

Syphilis is a highly infectious sexually transmissible infection (STI) caused by *Treponema pallidum*. The infection is systemic, usually involving ulcerative mucocutaneous lesions and rash in early phases, with a range of serious complications including cardiovascular and neurological disease in later phases [1], [2]. Syphilis in men who have sex with men (MSM) is of particular public health importance because of its potential to increase the risk of HIV transmission [3]. Syphilis can increase susceptibility to acquiring HIV infection and increase transmissibility [3], [4]. A recent retrospective cohort study in Australia demonstrated significant associations between a recent or past syphilis infection and HIV seroconversion [5].

In the last decade, rates of syphilis infections have increased among MSM in Europe, America and Australia [6]–[8]. MSM with HIV infection are disproportionately affected by syphilis. Enhanced surveillance in the same countries show that between 50% and 60% of infectious syphilis notifications in MSM are among men with HIV infection [6]–[8]. Community-based cohort studies in Australia have shown that the incidence of syphilis in MSM with HIV infection is five-times higher than the incidence in MSM without HIV (2.5 vs 0.5 per 100 person years) [9]. These differences reflect higher levels of risk behaviour among HIV-positive men [10].

Condom promotion has been used as a syphilis prevention strategy but has been largely ineffective in part because syphilis can be transmitted through oral sex, which rarely involves condom use. Also the marked long term sexual behaviour change required to reduce syphilis prevalence [11] has been reported as unacceptable to Australian gay men [12] and unlikely to be adopted as an individual and public health strategy [12].Optimal control of syphilis requires the early detection and treatment of infectious cases to prevent further transmission of infection. In addition to the individual benefits of increased testing, increasing the population coverage of syphilis testing could have considerable public health benefits. Mathematical modelling suggests that syphilis testing will have the greatest impact on reducing community transmission among MSM in Australia if two strategies are undertaken in combination: 90% of sexually active HIV-positive MSM are tested quarterly as part of HIV management checks, and 90% of MSM who have greater than 20 partners in six months get tested at least twice per year [13].The implementation of interventions based on these recommendations was forecast to reduce the number of syphilis diagnoses over a five year period [13].

In San Francisco, recommendations that HIV-infected MSM be screened for syphilis with every CD4 count or HIV viral load have been in place since 2008 [14]. In Australia, in 2005, clinicians were informally advised to conduct three-monthly syphilis testing in HIV-positive gay men as part of quarterly HIV management checks. This recommendation was more formally endorsed in the STI testing guidelines for gay men in 2010 [15] and form an integral part of the Australian National Gay Men's Syphilis Action Plan [13]. Despite these recommendations, little information are available on syphilis testing and clinical strategies used to integrate testing into HIV management checks. Such evidence can be used to re-calibrate mathematical models used to predict population benefits of testing, and guide clinical policies in Australia and overseas.

We describe the frequency of syphilis testing as part of routine HIV monitoring blood tests performed in HIV-positive MSM at a range of clinical sites in Australia over a five-year period, and differences by clinic syphilis testing policies.

## Methods

### Setting

The study was conducted in Sydney and Melbourne, the Australian cities in which the largest number of MSM reside. Seven clinics provided data for the study; three general practices which specialise in gay men's health, two sexual health clinics and two hospital HIV outpatient clinics (Table 1). These clinics were all in close proximity to each other and located in areas with the greatest concentrations of gay men.

**Table 1:** HIV viral load and syphilis testing policies at the seven clinics.

Clinic number	Frequency of HIV monitoring blood tests	Syphilis testing strategy	Test ordering system	Year syphilis testing policy introduced
1	3–6 monthly*	Opt-out	Electronic	April 2006
2	3–6 monthly*	Opt-out	Electronic	September 2006
3	3–6 monthly*	Opt-out	Manual	January 2008
4	3–6 monthly*	Opt-out/opt-in	Electronic	Same policy throughout
5	3–6 monthly*	Opt-in	Manual	Same policy throughout
6	3–monthly	Risk-based	Manual	Same policy throughout
7	3–6 monthly*	Risk-based	Manual	Same policy throughout

* 3 monthly and 6-monthtly if clinically stable.

### Definitions

In this paper *opt-out* refers to syphilis testing done automatically on all HIV-positive MSM unless a patient declines to have the test; *opt-in* means offering syphilis testing to HIV-positive MSM and conducting the test in those that agree (which may be related to their perceived risk); and *risk-based* involves assessing risk and then offering a syphilis test accordingly (eg. syphilis testing not offered to MSM who have not been sexually active since their last negative syphilis test).

### Testing policies

All seven clinics initially conducted routine HIV management checks (including CD4 counts and HIV viral loads) on HIV-positive patients quarterly, with six of the seven extending the visit frequency to six-monthly if the patient was clinically stable (Table 1).

Clinics 1–3 introduced opt-out syphilis testing as routine HIV monitoring blood tests, with the strategies implemented in April 2006, September 2006 and January 2008, respectively (Table 1). In Clinic 1 the strategy was first introduced in April, with all doctors adopting the system by late 2006. The approach involved syphilis testing being automatically included in HIV monitoring bloods. Two clinics used electronic requests while the other used a stamp on a paper request form. Clinicians had to purposely de-select the test from the batch of tests being requested if the testing was not required. The systems were simple and easy to set up. The electronic model was established in flexible patient management systems (PMSs) which allowed a single clinician to group tests in the pathology request section, and the group was available to all clinicians. The stamp model involved an administrative staff pre-stamping groups of pathology on request forms. All three systems had minimal to no costs.

The four other clinics used approaches which were implemented prior to the study period and did not alter during this time frame. Clinic 4 used a combination of approaches: three clinicians established an electronic opt-out system and the other clinician used an opt-in system where three groups of tests (CD4 count, HIV viral load; full blood count, liver function tests; syphilis) related to HIV monitoring were established in the electronic test request section of the patient management system, and the doctor ticked the appropriate ones depending on the nature of the consultation. Clinic 5 also used an opt-in approach where clinicians wrote syphilis testing on their pathology form when requesting other blood tests for HIV monitoring.

Clinics 6 and 7 undertook risk-based syphilis testing subject to the reported risk behaviour of the patient i.e. 3-monthly syphilis testing in those reporting higher risk behaviour such as multiple partners, attending sex-on-premises venues, or an STI on previous testing, as recommended in the testing guidelines [15]. One of these two clinics modified their patient management system to systematically record and alert the clinician to the required testing frequency based on the reported risk behaviour.

### Participants

Participants were men over the age of 18 years for whom at least one HIV viral load test was requested in 2006–2010 period. All clinics provided data for all men; an estimated >95% of their male HIV-positive clients were MSM. HIV viral load tests were used as an indicator of positive HIV status.

### Data collection

HIV viral load, syphilis testing and demographic data were collected retrospectively from the clinic or pathology laboratories servicing the clinic for the period of 2006 to 2010. Clinic 7 was unable to provide HIV viral load data for 2006–2007. A unique numerical identifier was available for each clinic to allow tracking of the same individual over time at the same clinic. No names or other personal identifiers were collated.

### Statistical methods

In each year, men were included if they had at least one HIV viral load test done. We calculated the mean and median number of syphilis tests conducted per patient per calendar year and the proportion undergoing none, one, two or ≥3 tests per year. As guidelines rely on standard HIV management checks, of which viral loads tests are a part, we then calculated the mean and median number of viral load tests conducted per patient per year, and the proportion undergoing one, two or ≥3 viral load tests per year. We also calculated the proportion of viral load tests that had a syphilis test conducted on the same day, and assessed the change in this outcome before and during introduction of syphilis test policies. All three outcomes were stratified by the various testing policies in Table 1.

Standard deviations (SDs) and interquartile ranges (IQRs) were calculated for means and medians respectively. We used a Chi-2 test to assess changes in syphilis testing frequency over time and between testing policies in Table 1.

In the three clinics which implemented opt-out strategies during the study periods, and had before and after periods, we compared the median monthly proportions of patients with same-day syphilis tests in the pre- and post-intervention periods using a ranksum test. Statistical significance was set at 0.05.

Analyses were conducted using Stata version 12.1 (StataCorp, College Station, Texas, USA).

Ethical approval was obtained from Alfred Health Human Ethics Committee Melbourne, St Vincent's Hospital Human Research Ethics Committee (HREC) Sydney and the University of New South Wales HREC Sydney. Informed consent was not obtained from the patients as the study involved a retrospective data analysis using anonymous data. This process was approved by all the ethics committees.

## Results

### Patients

In each 12-month period from 2006 to 2010 the total number of men having at least one viral load test ranged from 3131 to 3748; and by individual clinic it was highest at 627–767 per year, and lowest at 125–204 per year. The median age of the men was 44–45 years each year; and by clinic was highest at 61–63 per year, lowest at 37–38 per year, and 41–47 per year at the four other clinics.

### Syphilis testing frequency

The mean number of syphilis tests per man increased from 1.3 in 2006 to 2.2 in 2007 (p<0.01) and remained stable at 2.1 from 2008–2010 (Table 2).

**Table 2:** Frequency of syphilis tests in male regular clients, 2006–2010, by clinic.

Year	Syphilis testing frequency	Clinic							
		Opt-out				Opt-in	Risk-based		All
		1	2	3	4	5	6	7	
**2006**	0 (%)	8.7	41.3	36.5	2.4	10.4	50.7	-	27.9
	1 (%)	37.7	45.8	40.6	22.3	47.2	33.8	-	38.2
	2 (%)	24.7	11.5	16.3	35.3	30.4	11.0	-	19.2
	≥3 (%)	29.0	1.4	6.6	40.1	12.0	4.6	-	14.7
	Median (IQR)	2 (1–3)	1 (0–1)	1 (0–1)	2 (2–3)	1 (1–2)	0 (0–1)	-	1 (0–2)
	Mean (SD)	1.9 (1.5)	0.7 (0.7)	0.9 (0.9)	2.4 (1.3)	1.5 (0.9)	0.7 (1.0)	-	1.3 (1.3)
**2007**	0 (%)	3.2	3.1	13.8	1.0	11.4	29.8	-	9.4
	1 (%)	29.2	19.2	25.9	18.9	39.3	40.1	-	27.1
	2 (%)	25.1	30.8	26.3	26.9	31.4	16.6	-	26.0
	≥3 (%)	42.6	47.0	34.1	55.2	17.9	13.5	-	37.5
	Median (IQR)	2 (1–3)	2 (2–3)	2 (1–3)	3 (2–4)	1 (1–2)	0 (0–2)	-	2 (1–3)
	Mean (SD)	2.4 (1.4)	2.6 (2.2)	2.0 (1.4)	2.7 (1.4)	1.6 (1.1)	1.2 (1.2)	-	2.2 (1.7)
**2008**	0 (%)	5.5	3.8	4.0	3.8	16.9	29.0	36.1	12.3
	1 (%)	25.4	21.2	21.4	22.0	38.0	36.6	26.8	25.6
	2 (%)	27.1	30.4	19.9	25.5	26.1	16.1	20.4	24.0
	≥3 (%)	42.1	44.6	54.8	48.7	19.0	18.2	16.8	38.2
	Median (IQR)	2 (1–3)	2 (1–3)	3 (1–4)	2 (1–4)	1 (1–2)	1 (0–2)	1 (0–2)	2 (1–3)
	Mean (SD)	2.4 (1.3)	2.4 (1.3)	2.7 (1.4)	2.6 (1.4)	1.5 (1.1)	1.3 (1.3)	1.3 (1.3)	2.1 (1.4)
**2009**	0 (%)	3.4	4.4	2.3	2.0	7.0	30.0	32.6	11.5
	1 (%)	29.1	28.2	16.6	17.0	31.4	35.6	39.0	27.9
	2 (%)	25.5	33.4	21.8	23.3	30.3	21.8	17.5	24.6
	≥3 (%)	42.1	34.0	59.4	57.9	31.4	12.7	11.0	36.0
	Median (IQR)	2 (1–3)	2 (1–3)	3 (2–4)	3 (2–4)	2 (1–3)	1 (0–2)	1 (0–2)	2 (1–3)
	Mean (SD)	2.4 (1.4)	2.2 (1.3)	2.9 (1.4)	2.8 (1.4)	2.0 (1.2)	1.2 (1.1)	1.1 (1.2)	2.1 (1.5)
**2010**	0 (%)	2.5	5.0	2.4	3.7	3.4	36.5	54.3	14.2
	1 (%)	22.4	23.0	15.7	20.8	27.9	33.4	30.7	23.8
	2 (%)	28.7	37.1	22.5	22.1	29.9	18.6	9.7	24.8
	≥3 (%)	46.5	35.0	59.4	55.3	38.7	11.6	5.4	37.2
	Median (IQR)	2 (2–3)	2 (1–3)	3 (1–4)	3 (2–4)	2 (1–3)	1 (0–2)	0 (0–1)	2 (1–3)
	Mean (SD)	2.5 (1.4)	2.3 (1.2)	2.8 (1.3)	2.6 (1.4)	2.4 (1.2)	1.1 (1.2)	0.7 (0.9)	2.1 (1.4)

SD = standard deviation, IQR = interquartile range.

**Figure 1:**
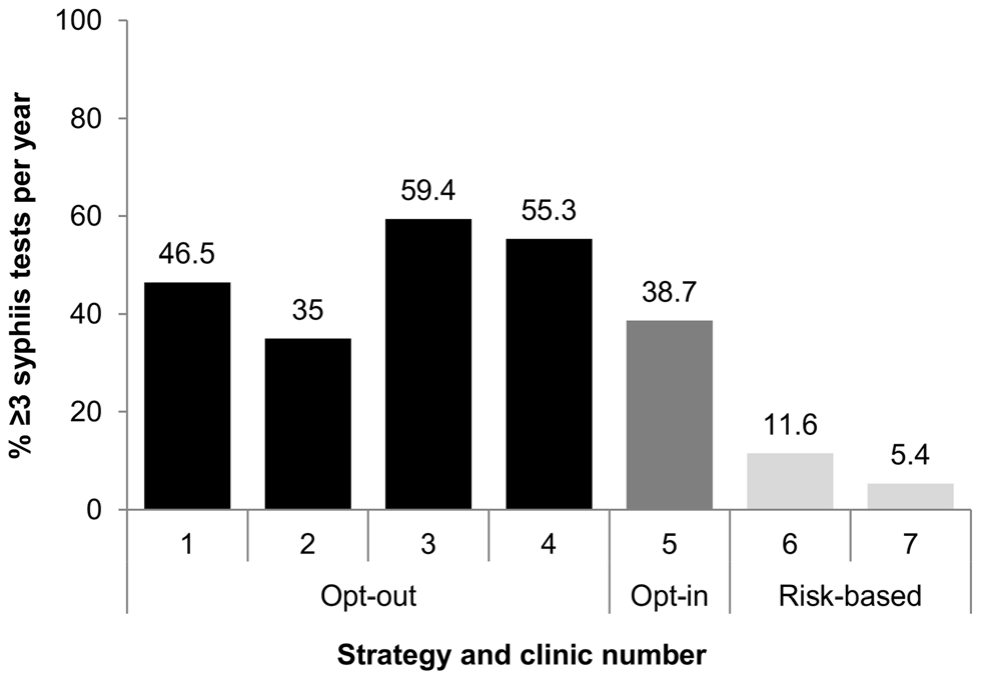
≥3 syphilis tests in 2010 by syphilis testing policy.

The proportion of men having ≥3 syphilis tests per year increased from 15% in 2006 to 36% in 2007 (p<0.01) and remained stable at 36–38% from 2008–2010 (Table 2). In 2010, the proportion of men having ≥3 syphilis tests in a year was highest in the clinics with opt-out strategies (48%, range per clinic: 35–59%), compared with opt-in (39%, p = 0.12) and risk-based strategies (8.4%, range per clinic = 5.4–12%, p<0.01) (Table 2, Figure 1).

In the same time period, the proportion of men having no syphilis test in a year decreased from 28% in 2006 to 9% in 2007 (p<0.01) and fluctuated between 11% and 14% in 2008–2010. In 2010, the proportion of men having no syphilis test in a year was lowest in clinics with opt-out strategies (3.4%, range per clinic = 2.4–5.0%), similar to opt-in (3.4%, p = 0.99) but substantially lower than risk-based strategies (46%, range per clinic = 36–54%, p<0.01).

### HIV viral load frequency

The mean number of HIV viral load tests per person remained stable at 2.6–2.8 per man per year between 2006 and 2010 (Table 3).

**Table 3:** Frequency of HIV viral load tests in regular clients, 2006–2010, by clinic.

Year	HIV viral load test frequency	Clinic							
		Opt-out				Opt-in	Risk-based		All
		1	2	3	4	5	6	7	
		%	%	%	%	%	%	%	%
**2006**	Once	40.9	26.2	22.7	21.6	40.8	21.0	-	28.6
	Twice	18.6	38.8	33.3	33.9	28.0	12.6	-	27.2
	Three or more	40.6	35.1	44.0	44.5	31.2	66.4	-	44.2
	Median (IQR)	2 (1–4)	2 (1–3)	2 (1–3)	1 (2–3)	2 (1–3)	3 (2–4)	-	2 (1–3)
	Mean (SD)	2.56 (1.6)	2.7 (1.1)	2.4 (1.2)	2.6 (1.4)	2.0 (1.0)	3.4 (1.8)	-	2.6 (1.4)
**2007**	Once	40.3	18.3	17.5	18.2	31.4	15.6	-	23.8
	Twice	18.9	26.9	19.9	26.3	30.0	14.2	-	21.8
	Three or more	40.8	54.7	63.0	55.6	38.6	69.9	-	54.4
	Median (IQR)	2 (1–3)	3 (2–4)	3 (2–4)	3 (2–4)	1 (1–3)	3 (2–4)	-	3 (2–4)
	Mean (SD)	2.3 (1.4)	2.8 (1.3)	3.0 (1.5)	2.9 (1.4)	2.3 (1.1)	3.3 (1.6)	-	2.8 (1.5)
**2008**	Once	36.8	16.5	21.5	19.4	23.9	16.8	56.9	27.3
	Twice	23.0	28.8	19.0	25.2	29.6	15.2	30.8	24.1
	Three or more	40.2	54.7	59.4	55.4	46.5	68.0	12.2	48.6
	Median (IQR)	2 (1–3)	3 (2–4)	3 (2–4)	3 (2–4)	2 (2–3)	3 (2–4)	1 (1–2)	2 (1–4)
	Mean (SD)	2.3 (1.3)	2.7 (1.3)	2.9 (1.4)	2.8 (1.4)	2.5 (1.2)	3.2 (1.5)	1.6 (1.0)	2.6 (1.4)
**2009**	Once	37.4	24.4	18.6	15.0	29.2	18.3	39.0	26.5
	Twice	21.3	32.6	19.4	19.5	29.7	17.9	22.3	23.2
	Three or more	41.3	43.0	62.0	65.5	41.1	63.8	38.8	50.2
	Median (IQR)	2 (1–3)	2 (2–3)	3 (2–4)	3 (2–4)	2 (1–3)	3 (2–4)	1 (1–3)	3 (1–4)
	Mean (SD)	2.4 (1.4)	2.5 (1.3)	2.9 (1.3)	3.1 (1.5)	2.3 (1.2)	3.1 (1.5)	2.4 (1.6)	2.66 (1.44)
**2010**	Once	29.6	18.8	16.4	18.1	26.0	16.4	41.6	23.0
	Twice	29.0	34.5	21.8	18.7	24.5	13.3	20.4	24.2
	Three or more	41.4	46.7	61.8	63.2	49.5	70.3	38.0	52.8
	Median (IQR)	1 (1–3)	2 (2–3)	3 (2–4)	3 (2–4)	2 (1–3)	3 (2–4)	1 (1–3)	3 (2–4)
	Mean (SD)	2.4 (1.3)	2.6 (1.2)	2.9 (1.3)	2.9 (1.4)	2.5 (1.3)	3.2 (1.5)	2.3 (1.5)	2.7 (1.4)

SD = standard deviation, IQR = interquartile range.

There was no significant trend in the proportion of men having ≥3 viral load tests per year over the 5-year period (44%–54%) (Table 3). In 2010, the proportion of men having ≥3 viral load tests per year was highest in the clinic with a three-monthly HIV viral load testing policy (70%) compared to 50% (range per clinic:38–63%, p<0.01) in the six other clinics with 3–6 monthly policies.

### Syphilis tests on the same day as HIV viral loads

**Figure 2:**
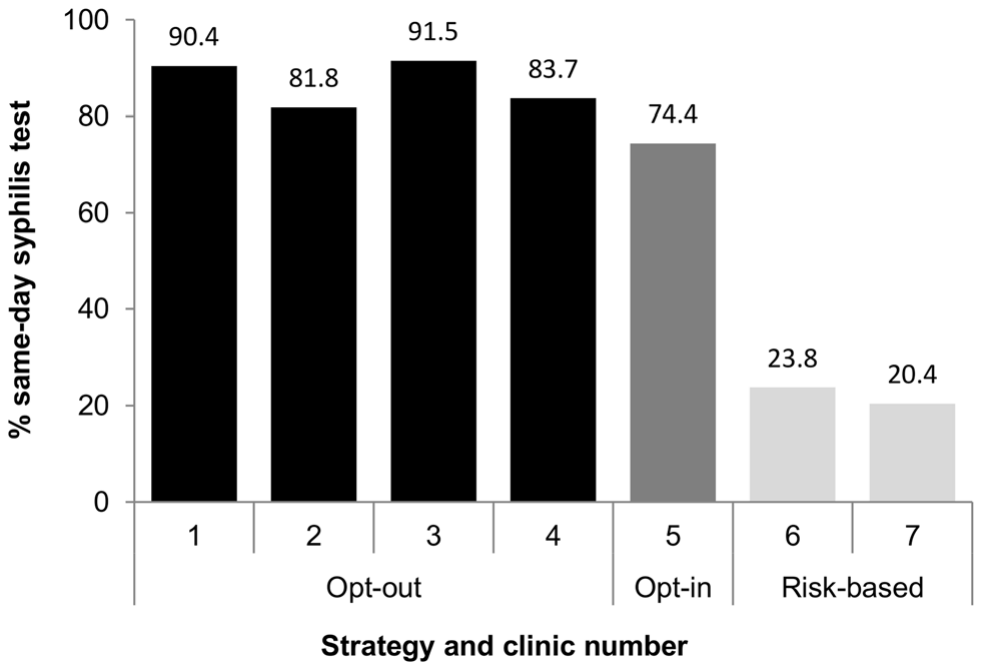
Same-day syphilis testing in 2010 by syphilis testing policy.

The proportion of men having viral loads with same-day syphilis test increased from 37% in 2006 to 63% in 2007 (p<0.01) and remained at 68–69% in subsequent years (Table 4). In 2010, the proportion of same-day syphilis tests was highest in clinics with opt-out strategies (87%, range per clinic = 84–91%) compared with opt-in (74%, p = 0.121) and risk-based strategies (22%, range per clinic = 20–24%, p<0.01) (Table 4, Figure 2).

**Table 4:** HIV viral load and syphilis test on same day in regular clients, by clinic 2006–2010.

Year	Clinic							
	Opt-out				Opt-in	Risk-based		All
	1	2	3	4	5	6	7	
	%	%	%	%	%	%	%	%
**2006**	67.5	29.0	32.2	88.7	62.6	12.2	-	43.3
**2007**	88.1	81.5	37.0	83.6	58.2	24.5	-	62.5
**2008**	85.8	82.6	79.6	85.3	51.8	31.1	33.0	69.2
**2009**	87.3	79.7	89.5	82.2	68.3	28.1	31.1	68.0
**2010**	90.4	81.8	91.5	83.7	74.4	23.8	20.4	69.4

**Figure 3:**
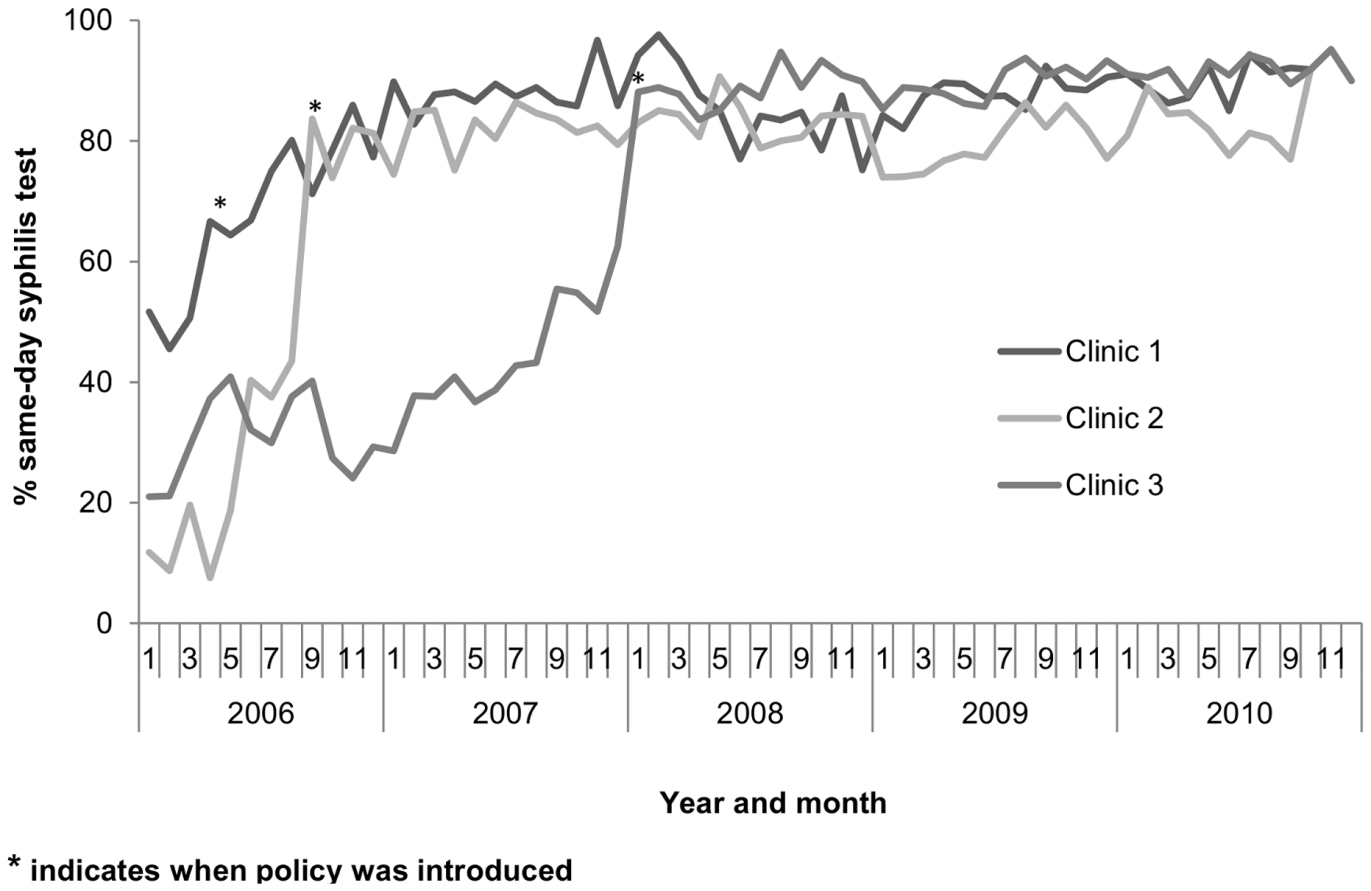
Same-day syphilis testing before and after introduction of opt-out strategies.

In Clinic 1, which introduced an opt-out strategy in April 2006, the median monthly proportion of HIV viral loads with a same-day syphilis testing was 58% (inter-quartile range(IQR) 0.51–0.67) in the pre-intervention period, compared with 88% (IQR:85–90%) in the intervention period (p<0.001). At Clinic 2, which introduced an opt-out strategy in September 2006, the median monthly proportion of HIV viral loads with same-day syphilis testing was 15% (IQR:9–20%) in the pre-intervention period, compared with 82% (IQR:78–84%) in the intervention period (p<0.001). At Clinic 3, which introduced an opt-out strategy in January 2008, the median monthly proportion of HIV viral loads with same-day syphilis testing was 37% (IQR:29–40%) in the pre-intervention period, compared with 89% (IQR: 87–90%) in the intervention period (p<0.001) (Figure 3).

## Discussion

To our knowledge this is the first study to compare syphilis testing frequency achieved through different clinic-based approaches to integrating syphilis testing into the care of HIV-positive MSM. Between 2006 and 2010 the frequency of syphilis testing in HIV-positive MSM increased considerably. Higher syphilis testing rates were found in clinics with opt-out and opt-in strategies, particularly the former, to integrate syphilis testing as part of HIV management checks. Importantly these strategies also decreased the proportion of men not tested for syphilis in a 12-month period.

The main strength of our study is the compilation of data from clinics in two different states and different types of clinics, which enabled us to compare the impact of various clinic policies. There are also a few limitations. We did not collect the syphilis testing results and the proportion that were asymptomatically infected as this had already been reported by Bissessor *et al*. based on data collated from one of the clinics in this study [16]. The clinics provided information on men only, not specifically MSM, but see a very small number of HIV-positive heterosexual men. Furthermore, it is possible that some men may have been in monogamous relationships and thus syphilis testing was deemed unnecessary by the clinician or patient. Many clinics in the study did not assess sexual behaviour routinely in their HIV-positive clients so this information was not available for the study. However the proportion of HIV-positive men in monogamous relationships is likely to be low, with a local survey showing 14% to 16% of the HIV-positive MSM reported having a regular partner only [17]. The intervention period in the analysis started from the time all clinicians were using the strategy. Therefore in clinics where uptake was more gradual due to different start dates by clinicians, the before period may have over-estimated the clinic activity, and in turn under-estimated the effect seen. Finally we had unique numerical identifiers for each clinic but did not have the patient names, so it is possible that some men attended more than one clinic and could be counted separately in two clinics. If these individuals had bloods done at both clinics it would reduce the apparent of HIV viral load testing or syphilis testing in a given year, but would not affect the proportion of tests where both viral load and syphilis tests were done.

Our findings highlight that opt-out strategies have successfully integrated syphilis testing into routine HIV management checks. Similar approaches have been reported in Melbourne (one of the clinics in this analysis) and the UK [16], [18], [19]. Opt-out HIV testing has also been used in MSM attending a sexual health clinic in the Netherlands where HIV testing was routinely performed unless the client refused, and HIV testing rates increased from 84% to 96% [20]. Similar approached have been used successfully for chlamydia testing among young people attending primary care clinics [21]. A recent systematic review demonstrated that of 15 interventions identified in the review, two which promoted the universal offer of a chlamydia test in young people had the greatest impact on increasing testing. These included providing urine jars to patients at registration which increased tested from 15% to 45% and doctors offering a test to all presenting young male clients prior to consultation from 4% to 29% [21].

However, while demonstrating success, the opt-out and opt-in strategies in our study were unable to increase syphilis testing to the recommended quarterly levels. As only about half of men were having three or more HIV viral loads tests per year, and about 80% of viral loads had syphilis tests conducted concurrently, then overall only 40% of men had quarterly syphilis testing. These findings are consistent with syphilis testing frequency self-reported by HIV-positive gay men in 2010 national behavioural surveys [22]. The lower than expected viral load testing reflects a shift in HIV monitoring policy during the study period, with 3–6 monthly viral load and CD4 cell count testing rather than 3-monthly for people with stable HIV infection, a change formally recommended in HIV monitoring guidelines since 2008 [23]. To achieve quarterly syphilis testing as suggested by the modelling to control syphilis, additional strategies may be needed. For example, in the Netherlands men could download referrals letters for syphilis testing and receive results online, rather than attending clinics [24].

In the two clinics which used risk-based testing in our study the frequency of testing was significantly lower, particularly at the clinic where there was also 3–6 monthly viral load testing, rather than three-monthly. Potential benefits of a risk-based strategy are appropriately targeted testing and reduced costs, but the strategy is also reliant on a risk assessment being done at each 3-monthly visit, as risk behaviour may change between the recommended testing period. The potential infections that could be missed with a risk-based strategy has been demonstrated at one of the clinics in this study that previously showed a substantial increase in the yield of early, asymptomatic syphilis; from 21% when they used a risk-based approach to 85% when they used an opt-out approach [16].

Although our analysis shows that the clinics have not achieved the 90% targets recommended from the mathematical modelling, there have been substantial improvements which may still lead to reductions in syphilis prevalence in the community. Between 2007 and 2010 in Melbourne there was a 35% decline in infectious syphilis notification in men overall.[Personal communication, Victoria Ministry of Health, 2011] Also in inner Sydney, syphilis notifications peaked in 2009, followed by a 22% decline from 2009 to 2010. [Personal communications, South Eastern Sydney Illawarra Public Health Unit, 2011] In both locations a similar decline was seen in both HIV-positive and negative men since 2008. Sentinel surveillance data from three of the four Melbourne clinics in this analysis has also demonstrated a decline in syphilis incidence in both HIV-positive and negative men coinciding with an increase in syphilis testing [25].

In conclusion, between 2006 and 2010 the overall frequency of syphilis testing in participating clinics has increased considerably. Higher syphilis testing rates were found in clinics with opt-out and opt-in strategies used to integrate syphilis testing during HIV management checks. The strategies were simple and implemented internally by the clinics. The systems should be considered at any service providing HIV management to MSM.
